# Supplementary material for: Are carbon emissions trading and green financial instruments synergistic? -Comprehensive quantitative research based on content analysis
Source: PLoS One. 2024 Mar 7;19(3):e0298601. doi: 10.1371/journal.pone.0298601 (PMC10919601; doi:10.1371/journal.pone.0298601)
Supplement: S1 Data — (ZIP) [file pone.0298601.s001.zip › Data/地级市Z_F工作报告.zip!/░▓╗╒/╕╖╤⌠╩╨/└┤╫╘╣1⁄2╓┌║┼í╛┬φ┐╦╩2╛▌═°í┐_2015.docx]

各位代表：

　　 现在，我代表市人民政府，向大会报告政府工作，请予审议，并请市政协委员和其他列席人员提出意见。

　　一、2014年工作回顾

过去的一年，在省委、省政府和市委的正确领导下，全市上下认真贯彻落实党的十八大和十八届三中、四中全会精神，坚持“稳中求进、进中求好、好中求快”工作总基调，科学把握大势，积极主动作为，奋力克难攻坚，有效保持了经济社会平稳较快发展。初步统计，全年生产总值1146亿元，比上年增长8.6%；财政收入180.2亿元，增长14.7%；城镇、农村常住居民人均可支配收入分别为21715元和8213元，增长9.2%和12.3%。

　 一年来，我们主要做了以下几个方面的工作：

（一）以激发活力为核心，着力加大改革力度。坚持从解决制约发展的突出问题入手，出台实施了一系列改革举措，取得了积极成效。行政体制改革有效展开，市政府职能转变和机构改革总体方案批准实施，工商、质监、食品药品监管体制改革进展顺利。行政审批制度改革初见成效，市级审批事项由148项减少到94项，精简36.5%，成为全省行政审批事项较少的市。市级涉企收费清单公布施行，收费项目由275项减少到145项，精简47.3%，位居全省前列。工商登记制度改革全面推行，新增各类市场主体3.7万户，位居全省第二位。金融体制改革步伐加快，8县市区农商行组建运营，交通银行阜阳分行正式开业，村镇银行实现县域全覆盖，颍元农业科技、温禾新材料等5家企业在“新三板”和省股权交易中心挂牌。市级投融资平台融资能力明显增强，县级投融资平台发展到19家。财税体制改革逐步深化，政府购买服务全面推开。农村综合改革试点工作扎实开展，临泉县、颍泉区土地确权登记基本完成。国有企业改革稳步推进，市糖酒公司、五交化公司等企业改制重组工作顺利完成。医药卫生体制改革深入实施，文化、教育、科技等领域改革也都取得了积极进展。

（二）以增量提质为目标，全力促进经济发展。坚持发展第一要务，全面落实稳增长各项政策措施，努力扩大经济总量、提升发展质量。工业经济较快增长，实现规模以上工业增加值445.6亿元，增长11.5%。规模以上企业发展到1258家，当年新增234家，位居全省首位。高新技术产业加快发展，实现产值224.5亿元，增长20.9%，增速居全省第三位。发明专利授权217件，增长69.5%。各类开发园区转型升级步伐加快，7个省级开发区扩区规划和临泉庐阳现代产业园获得省政府批准，阜合现代产业园区基础设施得到加强，拓展区建设全面启动，产业发展势头良好。现代农业加快发展，粮食总产连续七年超百亿斤，实现“九连增、十一连丰”，再次荣获“全国粮食生产先进市”称号。土地流转面积420万亩，流转率48.8%，高于全省平均水平7.7个百分点。经济作物面积扩大到453万亩，规模养殖比重达75%。规模以上农产品加工业产值632亿元，增长24.8%，增速居全省首位。农民专业合作组织发展到6440户，注册家庭农场1630户，太和县入选第三批国家现代农业示范区。森林增长工程深入实施，成片造林14.9万亩，超额完成年度目标任务。商贸市场繁荣活跃，实现社会消费品零售总额573.3亿元，增长13.2%。房地产业健康较快发展，完成投资215.7亿元，增长53.5%，增速居全省首位。万达广场、宝龙城市广场和临沂商城二期加快建设，大润发、家乐福等知名企业加快向县域布局。对外贸易较快增长，实现进出口总额16.1亿美元，增长17.9%。电子商务和快递业快速发展，电商主体发展到5000多家，快递行业业务收入增长55.6%。银行保险业稳健运行，年末金融机构存款余额2047.5亿元，贷款余额964亿元，分别增长17.1%和22.3%。

（三）以项目建设为抓手，努力扩大有效投入。坚持把扩大投资作为加快发展的主动力，项目谋划推进力度明显加大，全年实现固定资产投资805亿元，增长24.8%，增速居全省首位。商阜杭高铁即将开工，郑合高铁前期工作扎实推进，欣奕华新材料、天能电池二期等项目建成投产，金种子生态产业园、江汽重型卡车制造、阜阳煤基新材料和意大利生物质产业园建设进展顺利，阜阳卷烟厂易地技改、华润电厂二期项目获得批准。交通基础设施建设步伐加快，累计投入资金47亿元，开工建设国省干线公路350公里，升级改造县乡公路167公里，改造加固农村公路危桥125座。沙颍河航道整治工程进展顺利，阜阳机场改造工程基本完成。水利基础设施建设力度加大，累计完成投资15亿元。电力基础设施建设明显加强，新建改造变电站19所，新增输配电线路1270公里。切实加大招商引资工作力度，大力推进驻外招商、以商招商和以企招商，积极承接产业转移，全年引进市外资金970亿元，增长45.8%。

（四）以中心城市为引领，强力推进城乡建设。始终把加速新型城镇化进程作为大事来抓，深入实施中心城市带动战略，围绕建设大美阜城目标，高起点、大力度、全方位推进阜城建设与管理，全年完成投资160亿元，是上年的2.1倍，创历史最高。城市基础设施建设明显加强，阜颍路、北京路和人民西路等道路改造完成，西南大外环、东三环路加快建设，沙颍河船闸桥竣工通车，颍河向阳路大桥、东环路大桥开工建设。城南新区建设步伐加快，一期工程主干路网基本建成，市规划展示馆、要素大市场开工建设。阜城棚户区改造三年行动计划有效实施，一期工程进展顺利，完成征迁700万平方米，位居全省之首。坚持安置优先，开工建设各类保障房和安置住房63494套，基本建成20566套，超额完成年度目标任务。全面启动智能公交项目建设，新添置100台空调公交车，公共自行车系统投入运营。深入推进市容环境、交通秩序、农贸市场“三项整治”，大力实施园林绿化提升行动，新增绿地面积400万平方米。阜城的大建设大发展有效改善了人居环境，促进了经济增长，提升了对外形象，受到社会各界的好评。高度重视县城建设，着力扩张规模、完善功能，县城面貌明显改观，承载能力进一步增强。大力推进以城带乡，切实加强中心镇建设，美好乡村建设成效显著。深入开展“三线三边”综合整治，农村环境持续改善。

（五）以社会和谐为根本，倾力改善人民生活。坚持以人为本、民生为重，33项民生工程全面完成。高度重视就业再就业工作，城镇登记失业率控制在3%以内。深入开展“接您回家”系列活动，全年新增农村转移劳动力15.1万人，返乡创业3.3万人。扎实推进社会保障提标扩面，各项社会保险年度目标任务超额完成。加强住房公积金管理工作，累计归集资金106亿元，发放贷款73亿元。深入实施阜城基础教育设施三年提升计划和阜阳医疗资源建设三年发展计划，累计完成投资40亿元，阜城18所学校建成使用，15所学校正在加快建设。颍泉、颍东区义务教育均衡发展通过国家评估认定，阜阳幼儿师范高等专科学校设置工作通过省级专家评审。全市8个医院项目建成运营，市人民医院新区和安医大附属阜阳医院等18个项目加快推进。深入实施文化惠民工程，阜南、临泉、颍上、太和县被命名为“中国民间艺术之乡”。积极发展体育事业，竞技体育水平进一步提高，我市运动员赵福祥获得仁川亚运会武术散打冠军。深入开展精神文明创建活动，再次荣获“安徽省文明城市”称号。切实加强生态文明建设，耕地保护目标任务全面完成。大力实施大气污染防治“颍淮蓝天工程”，秸秆禁烧和综合利用工作成效显著，空气质量位居全省第三位，全国农业循环经济现场会在我市召开。坚持计划生育基本国策，低生育水平持续稳定。组织实施新一轮扶贫开发攻坚计划，认真解决“老字号”群体生活困难问题，以船为家渔民上岸安居工程顺利完成。强化食品、药品和生产安全监管，安全生产形势总体平稳。切实加强信访工作，累计化解信访积案659件。深入推进社会治安综合治理，禁毒攻坚战、整体战取得初步成果，社会大局保持和谐稳定。积极开展强武阜阳建设，认真做好武警、人防和民兵预备役工作。加强基层社区建设，第九届村委会换届工作全面完成。妇女、儿童和老龄、老干部工作进一步加强。红十字、社会慈善及残疾人事业稳步发展。民族、宗教、档案、地方志、地名、气象、地震、外事、侨务、港澳台等工作也都取得了新的进展。

（六）以转变作风为重点，大力加强自身建设。坚持为民务实清廉主题，扎实开展党的群众路线教育实践活动，自觉践行“三严三实”，集中整治“四风”积弊，着力解决人民群众关心的热点难点问题。深入推进依法行政，坚持市政府常务会议学法制度，启动编制政府权力清单和责任清单，切实规范行政行为。自觉接受市人大法律监督和市政协民主监督，办理人大代表建议127件、政协委员提案199件。认真做好市长热线和网络问政工作，大力推进政府信息公开。全面推行政务服务标准化，行政审批电子监察系统建成使用。严格执行廉洁从政各项规定，开展公务用车、办公用房专项清理，市级“三公”经费下降18.2%，会议压缩23.7%，文件减少16.3%。加大公共资源交易监管力度，强化行政监察和审计监督。完善政府目标管理绩效考核体系，开展重点领域政策落实专项督查，提高了工作效能，保障了政令畅通。

在肯定成绩的同时，我们也清醒地看到，我市经济社会发展中还存在一些困难和问题：一是发展不足、发展不快和人均水平不高的问题依然突出，战略性新兴产业增量不大，部分传统行业运行困难。二是企业融资难、融资贵问题比较明显，征迁安置工作相对滞后，资源环境约束加剧，要素制约日益凸显。三是社会事业发展滞后，交通、水利、电力、教育、卫生等基础设施相对薄弱，基本公共服务均等化水平仍然不高。四是信访维稳工作压力较大，食品、药品和生产安全监管形势复杂，禁毒工作任务艰巨，农村治安防控体系亟待加强。同时，少数干部安于现状、为官不为，敢于担当、主动作为的意识不强，“四风”问题和消极腐败现象还不同程度地存在，发展环境有待进一步优化。对此，我们一定高度重视，采取有力措施，认真加以解决。

各位代表！过去的一年，在面对诸多不利因素和挑战的情况下，我们能够取得这样的成绩，确实来之不易。这是省委、省政府和市委坚强领导的结果，是市人大、市政协大力支持的结果，是全市上下团结一心、奋力拼搏的结果。在此，我代表市人民政府，向广大工人、农民、知识分子、干部、离退休老同志，向驻阜解放军指战员、武警官兵和政法干警，向各民主党派、工商联、无党派人士、各人民团体和社会各界人士，向所有关心支持阜阳建设与发展的同志们、朋友们，表示衷心的感谢！

　　二、2015年工作安排

2015年是全面深化改革的关键之年，是全面推进依法治市的开局之年，也是全面完成“十二五”规划的收官之年，做好今年各项工作意义重大。当前，我国经济发展已进入速度变化、结构优化、动力转换的新常态，新常态带来新变化，新变化蕴含新机遇，新机遇昭示新发展。我市既有着人口众多、资源禀赋、交通区位、市场广阔等诸多优势，又有着国家中部崛起、中原经济区和长江经济带建设以及省加快皖北振兴等重大机遇，发展的空间、潜力和韧劲都很大。同时，经过多年努力，我市政治生态和发展环境明显优化。只要全市上下顺势而上、奋勇争先、扎实苦干，就一定能够开创经济社会发展新局面。

今年政府工作的总体要求是：认真贯彻党的十八大和十八届三中、四中全会精神，全面落实市委四届八次全会和全市经济工作会议部署，紧紧围绕“皖北争一流、全省赶平均、同步达小康”的目标，积极适应经济发展新常态，坚持质量、效益与速度、总量并重，注重拓展转型发展新空间与放大传统产业优势相结合，深入推动改革创新，持续扩大有效投入，大力推进新型工业化、信息化、城镇化和农业现代化进程，切实加强民生保障，促进经济持续健康较快发展和社会和谐稳定。

　　经济社会发展主要预期目标是：全市生产总值增长8.5%左右，财政收入增长12%，固定资产投资增长20%以上，规模以上工业增加值增长12%，社会消费品零售总额增长12%，进出口总额增长13%，城镇、农村常住居民人均可支配收入分别增长10%和13%，居民消费价格涨幅控制在3%左右，城镇登记失业率控制在4%以内，全面完成“十二五”节能减排任务。

　　实现上述目标，重点抓好8个方面的工作：

　　（一）全面深化重点领域改革

　　切实把改革作为破解难题、促进发展的关键，突出问题导向，狠抓重点突破，最大限度激发市场活力和社会创造力。

　　深入推进行政体制改革。坚持把简政放权作为全面深化改革的突破口，承接落实好省取消下放的行政审批事项，继续清理减少行政审批事项，全面清理非行政许可审批，努力以政府权力的“减法”换取市场活力的“加法”。认真实施涉企收费清单，全面推行政府权力清单和责任清单。深入推进行政审批“两集中、两到位”，建立政府权力清单运行平台，简化权力事项前置审批条件，加强事中事后监管。规范完善招投标管理制度，推进市、县一体化公共资源交易平台建设。加快政府职能转变和机构改革，积极推动事业单位分类改革。

　　深入推进农村综合改革。在总结试点经验的基础上，全面展开农村土地确权登记颁证工作，引导承包经营权有序流转，促进农业适度规模经营。加快建立城乡统一的建设用地市场，积极探索城乡建设用地增减挂钩指标跨区域流动，开展工矿废弃地复垦利用试点。加快建设农村产权流转交易市场，深化小型水利工程产权制度改革，稳步开展农民住房财产权抵押、担保、转让试点，探索宅基地有偿退出机制，盘活农村存量建设用地，逐步把沉睡的资产变成流动的资本。

　　深入推进财税金融改革。进一步完善税收征管机制，促进财政收入稳定增长。健全预算管理制度，实行预算绩效评价结果与预算安排、资金分配挂钩，完善县级基本财力保障机制。加强政府债务管理，依法适度举债融资，建立规范的举债融资机制。全面深化农村金融综合改革，扎实推进村级服务站和农村信用体系建设，着力提升农村商业银行和村镇银行资本实力与服务能力。推进政策性农业保险扩面提标，全面推行保单质押贷款。积极引进商业银行在我市布点，支持民间资本发起设立中小银行等金融机构，加快政策性融资担保体系建设。

　　深入推进国有企业改革。以发展混合所有制经济为方向，坚持分类指导、一企一策，加快国有企业改革步伐。支持有条件的企业改组为投资公司或资本运营公司，鼓励和规范投资项目引入非国有资本参股。完善现代企业制度和法人治理结构，健全国有企业负责人激励约束机制。进一步加强国有企业监管，提高国有资本配置和运行效率，实现效益最大化。

　　（二）千方百计促进经济发展

　　始终把产业发展作为经济工作的基础和核心，统筹做大工业、做强农业、做活三产，促进三次产业协调发展。

　　切实加大工业突破力度。牢固树立工业立市、工业强市理念，大力促进工业经济较快增长。围绕扩大工业经济增量，持续加大工业投入，以“高、大、新、特”为方向，大力推进产业招商、集群招商和以商招商，着力引进一批成长性好、带动性强、竞争优势明显的大项目。引导支持现有企业发挥自身优势，拉长拉高产业链，推动上下游产业集聚发展。深入开展与央企、知名民企对接合作，促进更多优质企业和项目落户阜阳。围绕扩张工业经济存量，立足支柱产业和规模企业，加快改造升级，推动优势产业做大做强，促进骨干企业提质增效，努力打造千亿元产业、百亿元企业。加快金种子生态产业园、江汽重型卡车制造、阜阳煤基新材料产业园等重点项目建设，推动阜阳卷烟厂易地技改、华润电厂二期项目和意大利生物质产业园及早开工。发展壮大小微企业，完善扶持政策，加大培植力度，力争全年新增规模以上工业企业200家以上。围绕做大工业发展平台，加快推进开发园区扩区升级，加强基础设施建设，不断提升综合承载能力。重视做好阜阳经济技术开发区创建国家级开发区工作，大力支持阜合现代产业园区和临泉庐阳现代产业园加快建设。

　　切实加快发展现代农业。紧紧围绕“规模、结构、转化、合作”，着力转变农业发展方式，推进“生产导向”向“消费导向”转变，走出一条产出高效、产品安全、资源节约、环境友好的现代农业发展之路。深入推进粮食生产“三大行动”，依靠科技，提高单产，稳定总产，为国家粮食安全作出贡献。加强粮食收储能力建设，实施粮食危仓老库维修改造和农户科学储粮工程。按照近郊发展都市农业、远郊发展特色农业的思路，大力调整农业结构，扩大高效经济作物面积，建设一批特色农业、品牌农业、休闲农业示范乡镇和示范村。深入实施新一轮畜牧业升级计划和“菜篮子”工程，推进水产业健康养殖。充分发挥农业“接二连三”的作用，引导城市工商资本进入现代农业、整合产业链，支持农业产业化联合体实现产业、要素、利益联结，大力推进农副产品就地加工转化，推动“百亿粮仓”向“千亿厨房”转变。积极构建新型农业经营体系，启动实施新型职业农民培育计划，扶持家庭农场、农民专业合作社持续健康发展。切实加强高标准基本农田建设，深入实施小型水利工程改造提升行动计划，促进农技、农机、农艺结合，提高气象为农服务能力。

　　切实提升现代服务业水平。按照“立足阜阳、服务皖北、辐射中原”的战略定位，加快培育大市场，发展大贸易，搞活大流通，拓展新业态。认真落实促进消费增长的政策，将“营改增”范围扩大到建筑业、房地产业、金融业和生活性服务业等领域，实施养老健康家政、信息、旅游、住房、绿色、教育文化体育等6大领域消费工程，推动研发设计、信息技术等生产性服务业蓬勃兴起，促进电商、网购和快递业融合发展。加快建设阜阳青年电子商务产业园。深入实施城市商业网点规划，提升“新网工程”建设水平，进一步健全城乡市场体系。大力发展现代物流业，优化仓储和配送中心布局，推进阜阳公路物流港、粮食现代物流园等物流园区建设。认真落实加快旅游业发展的政策措施，实施乡村旅游富民工程，促进旅游休闲消费。深入实施外贸企业孵化工程，加强出口示范基地建设，争取设立海关特殊监管区，提升通关一体化和投资贸易便利化水平，不断扩大对外贸易。

　　（三）坚定不移扩大有效投资

　　牢固树立抓项目就是抓经济、抓投资就是抓发展的理念，持之以恒扩大有效投入，不断放大投资对经济增长的乘数效应。

　　高起点谋划编制项目。瞄准国家产业政策导向，立足产业基础，着力在基础设施、棚户区改造、生态建设、新兴产业和公共服务等领域，高标准谋划编制一批打基础、管当前、利长远的重大项目。加大项目申报推介力度，扎实做好前期准备和争取工作，努力以资源换项目、以市场换项目，力争更多的项目进入国家和省计划的盘子。同时，切实加强力量，集中时间，认真做好“十三五”规划编制工作。

　　全方位拓宽投资渠道。突出招商引资主渠道作用，加强驻外招商工作，切实提高招商实效，确保全年引进市外资金增长25%以上。进一步加强政银企对接，引导金融机构创新金融产品和服务方式，促进信贷投放规模持续增长。引导符合条件的企业上市融资、在“新三板”和股权交易市场挂牌。加大土地征迁收储力度，加快出让步伐，努力盘活土地收益。积极争取政策性投资，推广运用政府与社会资本合作（PPP）等模式，调动社会投资的积极性。

　　大力度推进项目建设。按照经济工作项目化、项目工作责任化的要求，全面推进项目建设提质提效，切实提升项目开工率、竣工率和运行绩效。全年计划实施亿元以上项目317个，年度计划投资495亿元。加快重点项目建设步伐，大力支持商阜杭、郑合高铁建设，认真做好阜阳至淮滨高速等项目前期工作，确保阜阳至颍上、太和至界首和阜阳至杨楼孜快速通道年内竣工通车，建成国省干线公路115公里，升级改造县乡公路210公里。扎实推进沙颍河航道整治工程，开工建设颍上复线船闸，积极争取一批项目纳入引江济淮工程。加强电力基础设施建设，新建和改造变电站19所、输配电线路2000公里。

　　（四）加速推进城市建设步伐

　　立足打造皖北区域性中心城市，抢抓国家新型城镇化试点机遇，加快构建“四位一体”的新型城镇化体系。

　　进一步做大做美阜城。阜城是我市城市体系的龙头，是经济发展的重要舞台、人民生活的重要平台、对外开放的重要窗口，必须把大美阜城建设摆上更加突出位置，坚持不懈地抓紧抓实抓好，努力完成340亿元年度投资计划。突出做大城市规模，适时开工一批重点基础设施项目，完成阜王路南延、涡阳路北延和颍上南路等工程，加快建设东三环、西南大外环和颍河向阳路大桥、东环路大桥，开工建设解放北路泉河桥、颍州路颍河桥。重点抓好城南新区和阜合现代产业园区建设，完成城南新区一期主干道路工程，实施水环境综合整治，开工建设市图书馆、博物馆、科技馆、文化馆、工人文化宫和大剧院，加快新华图书城和全民运动中心建设进度，做好二期规划设计工作。统筹实施好颍河综合治理、三角洲东岸人居环境综合治理和阜阳职教园区等重点项目。突出提升城市内涵，健全公共服务设施，不断完善城市功能。深入实施棚户区改造三年行动计划，全面加快推进步伐。按照两年内解决历史欠账、今后不留新账的目标，加快推进保障性安居工程和安置小区建设，积极探索货币化安置。大力发展公共交通事业，加快编制城市轨道交通规划，今年再增加100台空调公交车。开展城市地下管网普查和提升改造，新建一批小广场、小游园和停车场，阜城三区年内各建成一所青少年活动中心。突出加强城市管理，实行全面排查、综合整治、立体提升、精细管理，坚定不移把“三项整治”工作向纵深推进。加大园林绿化力度，坚持拆墙透绿、拆违增绿、见土必绿，突出加高、加密、加绿、加彩，努力塑造四季常青、五彩缤纷的城市形象。全面推行市场化运作，创新城市管理机制，不断提升城市综合管理水平。

　　进一步加快县城建设。按照中等城市标准，深入开展县城建设管理提升行动。坚持规划先行，积极探索“多规合一”，促进县城建设科学合理有序开展。重点加强市政公共服务设施配套，积极引进建设一批城市综合体、商贸市场和医院学校，提升县城综合承载能力。注重加强县城管理，大力开展硬化、绿化、净化、美化、亮化专项行动，切实改善城市形象，着力打造经济繁荣、环境优美、特色鲜明、社会和谐的美好县城。

进一步推进城乡统筹。扎实开展重点镇扩权增能试点，先行抓好20个特色集镇建设。加快户籍制度改革，完善相关配套措施，促进农业转移人口市民化。高度重视美好乡村建设，着重抓好重点示范村推进工作。以“三线三边”为突破口，深入推进城乡环境综合整治，研究建立长效机制，逐步实现常态化、规范化。扎实推动“物的新农村”与“人的新农村”齐头并进，促进基础设施向农村延伸、公共服务向农村覆盖、现代文明向农村辐射。

　　（五）积极有效推动转型升级

　　以国家创新型省份建设为契机，大力推进转型发展、创新发展，有效激发创业活力，加快培育和形成新的竞争优势。

　　深入实施创新驱动战略。以科技创新推动产业结构升级，培育壮大战略性新兴产业，实施生物医药、电子信息、装备制造、新能源和新材料等创新工程。运用高新技术改造提升传统产业，加快产品升级，培育自主品牌，着力推动传统产业向中高端迈进。加强企业自主创新能力建设，深化产学研合作，全面落实知识产权质押融资政策，发展壮大一批创新型企业和高新技术企业，支持开发园区创建省级高新技术产业开发区。加强创新型人才队伍建设，多措并举培养人才，加大力度引进人才，优化环境用好人才。大力促进信息化与新型工业化、城镇化和农业现代化深度融合，统筹推进国家智慧城市和信息惠民试点城市建设，加强城市公共服务平台、新型信息服务平台和云计算中心建设，推动农业物联网技术运用。

　　广泛开展全民创业行动。全面落实鼓励全民创业各项政策措施，深入推进商事制度改革，优化创业环境，完善服务体系，激发全社会创新创造活力。加快建设创业孵化基地，推动科技创业中心建设，支持民间资本发起设立产业投资基金和股权投资基金。鼓励引导大众创业，支持返乡农民工、高校毕业生和企业下岗职工等群体创业就业，以就地创业带动就地就业，在颍淮大地掀起大众创业、万众创新的热潮。

　　大力推动特色经济发展。围绕富民强县，引导支持各地立足自身条件和产业基础，发挥比较优势，推进错位发展，培育特色经济，建设一批布局合理、产业集聚、用地集约的开发园区。大力发展“一镇一业”、“一村一品”，打造一批具有本土特色的工业大镇、农业强镇、商贸重镇和旅游名镇，加快形成主业突出、特色鲜明、活力迸发的区域经济发展新格局。

　　（六）持续加强生态阜阳建设

　　主动顺应人民群众对美好生活的新期待，推进绿色低碳循环发展，努力让空气更清新、水质更清澈、环境更清洁。

　　加快生态环境治理步伐。深入推进大气污染防治“颍淮蓝天工程”，加强工业废气、城市扬尘和机动车尾气等专项整治，加快淘汰黄标车和老旧车。科学有效加强秸秆禁烧，进一步提升综合利用水平，促进PM10平均浓度继续下降，努力减少雾霾天气。启动实施水污染、土壤污染防治行动计划，推进重污染河流治理，加快城镇污水处理设施建设。坚持不懈地推进绿色阜阳建设，深入实施森林增长工程，大力开展全民植树行动，积极创建森林城市、森林城镇、森林村庄。

　　加大资源循环利用力度。坚持以资源高效利用和循环利用为核心，以“减量化、再利用、资源化”为原则，深入实施种植养殖循环发展、有色金属综合利用、煤化工节能技术应用等“十大重点工程”，推动循环经济向更高层次、更广领域迈进。加快推进重大节能环保技术产业化，发展太阳能、地热能、生物质能等新能源，扩大天然气利用规模。认真实施节能减排低碳发展行动计划，加强节能评估，强化合同能源管理，确保能源消费强度和总量控制目标的实现。

　　加强生态文明制度建设。认真宣传贯彻新修订的《环境保护法》，切实增强全民环保意识。实行最严格的耕地保护和水资源管理制度，依法处置闲置和低效利用土地，合理开发利用矿产资源，加强采煤塌陷区综合治理。强化水资源节约利用和水源地保护，严格控制中深层地下水开采，提高中水利用率。加强节能减排制度建设，推进环境污染第三方治理。完善资源环境承载能力监测预警机制，落实生态环境损害责任终身追究和赔偿制度。支持颍上八里河、颍州西湖创建国家自然保护区，支持阜南王家坝、颍东东湖和界首泉河“两湾”创建国家湿地公园。

　　（七）尽心尽力改善民计民生

　　按照守住底线、突出重点、完善制度、引导舆论的基本思路，织牢织密民生安全网，大力推进基本公共服务均等化。

　　切实抓好民生工程。按照省政府部署，深入实施33项民生工程，强化民生工程绩效管理，扩大惠泽民生的效益。加强农村饮水安全工程建设，提高集中供水覆盖率。扎实做好就业再就业工作，重点抓好农村转移劳动力、高校毕业生、退役军人和城镇就业困难人员等群体就业，持续深入开展“接您回家”系列活动，鼓励引导农民工就地就业、返乡创业。进一步完善社会保障体系，推进社会保险扩面征缴，重点做好农民工、灵活就业人员等群体参保工作，加快社会保障“一卡通”建设。认真实施机关事业单位养老保险制度改革，逐步提高基本工资标准。切实加大社会救助力度，完善重特大疾病医疗救助政策，健全社会救助体系。深入实施扶贫开发，全面推进科学扶贫、精准扶贫、教育扶贫，推动扶贫工作取得新进展。

　　统筹发展社会事业。落实教育优先发展战略，开工建设阜阳七中等10所学校。深入推进教育体制综合改革，促进义务教育均衡发展。大力发展职业教育，加快构建以就业为导向的现代职业教育体系。认真做好阜阳幼儿师范高等专科学校筹建工作，支持阜阳师范学院创建阜阳大学。加强医疗基础设施建设，确保圆满完成年度计划任务。全面启动城市公立医院综合改革，加强医疗联合体建设，发展中医药事业。认真落实“单独两孩”政策，稳定适度低生育水平。广泛开展全民健身运动。健全农村留守儿童、妇女、老年人关爱帮扶体系，完善残疾人民生和权益保障制度。加快发展养老产业，提升养老服务水平。支持工会、共青团、妇联和关工委、老龄委工作。积极推进档案、地名、地方志、红十字和社会慈善事业，进一步做好民族、宗教、外事、侨务、港澳台等各项工作。

　　大力推动文化繁荣。实施公民道德建设工程，广泛宣传社会主义核心价值观，弘扬优秀传统文化，开展“阜阳最美人物”系列评选活动，以“阜阳好人”现象树立“阜阳好人”形象。加快文化体制改革，促进文化产业优化升级，推动文艺精品创作和文化品牌建设。大力开展精神文明创建活动，推进文明城市、文明村镇和文明单位创建工作。深入实施文化惠民工程，办好颍淮群众文化大舞台。加快构建现代公共文化服务体系，加强城市社区、乡镇综合文化服务中心和中心村农民文化乐园建设，增强文化整体实力和竞争力。

　　（八）切实维护社会和谐稳定

　　注重运用法治思维和法治方式化解矛盾、处理问题，不断创新社会治理，更好地保障人民安宁、社会安定。

　　扎实推进法治阜阳建设。大力开展法制宣传教育，深入实施“六五”普法，深化“法律六进”活动，率先把美好乡村建成法治示范村。加快公共法律服务体系建设，加强困难群众法律援助。大力推行“阳光信访”，严格落实领导干部接访约访下访制度，健全依法维权和矛盾纠纷调处机制。加强人民调解组织建设，畅通群众利益协调和权益保护渠道，切实维护群众合法权益。加快社会诚信体系建设，树立“信用阜阳”的良好形象。

　　加快健全公共安全体系。严格落实安全生产责任制，深入开展经常性的重点领域安全隐患排查和专项整治，加强人员密集场所和公共场所安全管理，促进安全生产形势持续稳定好转。强化食品药品安全监管，建立健全全程监管和问责机制，保障公众饮食用药安全。进一步完善应急管理预案，加强应急平台和应急专业队伍建设，提高气象、地质、地震灾害防御能力。

　　全面提升社会治理水平。深入推进平安阜阳建设，健全立体化、数字化社会治安防控体系，开展突出治安问题专项整治，严厉打击各类违法犯罪活动，坚决打好禁毒攻坚战，切实维护社会和谐稳定。积极推进多层次多领域依法治理，深化社区管理体制改革，增强居民自治功能，做好社区矫正工作。依法加强网络社会管理，推进社会组织直接登记。

　　支持驻阜部队建设，加强国防动员、后备力量和人民防空工作。高度重视国家安全和保密工作。认真做好双拥优抚安置工作，推进军民融合深度发展，巩固军政军民团结大好局面。

　　三、加强政府自身建设

新形势、新任务对政府工作提出了新的更高要求。各级政府必须深刻认识新常态、主动适应新常态、积极引领新常态，全面加强自身建设，切实提高行政效能和施政水平。

（一）坚持勤奋学习，不断增强领导发展能力。切实把学习作为工作之基、能力之本、水平之源，认真学习党的十八大和十八届三中、四中全会精神，学习习近平总书记系列重要讲话精神，始终做到在思想上、政治上、行动上同党中央保持高度一致。坚持理论联系实际，主动学习政策、研究政策、用好政策，善于把上级精神、外地经验同本地实际结合起来，学会在逆势中寻求机遇、在风险中破解难题、在政策中拓展空间，全面提升研判形势、驾驭全局、推动发展的能力。进一步强化问题导向、底线思维，善于观大势、谋大事，积极探索新常态下的新思路、新措施、新方法，着力激发新活力、培育新动力、促进新发展。

（二）坚持依法行政，切实加快政府职能转变。牢固树立法治意识，时刻牢记法律红线不可逾越、法律底线不可触碰，大力推动学法、尊法、用法、守法，善于运用法律手段管理经济和社会事务。加强法治政府建设，严格按照法定权力和程序履行职能，做到法定职责必须为、法无授权不可为。着力规范政府系统决策行为，健全重大决策终身责任追究制度及责任倒查机制，推进决策科学化、民主化、规范化。完善行政执法管理，建立行政处罚裁量权基准制度。自觉接受人大及其常委会法律监督，依法执行人大决议、决定，认真办理人大代表议案、建议。主动接受政协民主监督，认真办理政协委员提案，广泛听取各民主党派、工商联和无党派人士的意见，围绕科学民主决策加强协商。自觉接受司法监督、舆论监督和公众监督，推进政务公开、办事公开，确保权力在阳光下运行。

（三）坚持执政为民，着力提升机关行政效能。巩固拓展党的群众路线教育实践活动成果，完善领导干部直接联系和服务群众机制，自觉走基层、听民声、接地气，做到问政于民、问需于民、问计于民。大力整治“四风”，推动作风建设常态化。强化责任担当意识，始终把干事创业作为最大责任，坚决纠正懒政、怠政和为官不为的现象，切实提升执行落实能力，提高一次部署完成率。加大经济环境治理力度，加强行政效能建设，努力营造良好的发展环境。强化政务督查，严格行政问责，做到推动工作只划“句号”、不划“逗号”，确保政令畅通、令行禁止。

（四）坚持严以律己，扎实推进反腐倡廉建设。认真履行党风廉政建设主体责任，坚持一岗双责、守土有责，完善惩治和预防腐败体系，推进反腐倡廉制度创新，加强行政监察和审计监督，坚决查处各类违法违纪案件。严格执行中央八项规定、国务院“约法三章”和省、市相关要求，加快公务用车制度改革，严控“三公”经费支出。全体政府工作人员尤其是领导干部要严守政治纪律、政治规矩，始终牢记“两个务必”，时刻绷紧廉洁自律这根弦，自觉提高拒腐防变的能力，努力当好人民满意的公仆。

各位代表！适应新常态任务艰巨，谋求新发展使命光荣！让我们紧密团结在以习近平同志为核心的党中央周围，在市委的坚强领导下，开拓进取，奋力拼搏，扎实苦干，为全面完成今年各项任务、顺利实现“十二五”规划目标而努力奋斗！
